# A Case of Tumour in the Orbit

**Published:** 1887-09

**Authors:** F. Richardson Cross

**Affiliations:** Surgeon to the Bristol Eye Hospital, Ophthalmic Surgeon to the Bristol Royal Infirmary


### A CASE OF TUMOUR IN THE ORBIT.

By F. Richardson Cross, M.B. Lond., F.R.C.S.; Surgeon to the Bristol Eye Hospital, Ophthalmic Surgeon to the Bristol Royal Infirmary.

The following case is unusual in its clinical history. I am of opinion that it is not any form of gumma, nor a hydatid; it can scarcely be looked upon as inflammatory, though its histology is probably similar to granulation tissue-formations. There can be no longer any suspicion of its malignant nature.

Thomas G., aged 35, in 1884 was "out of sorts," and was able to take very little exercise. He considers that his illness commenced with a chill taken during his work in a newly-built washhouse. His health varied for a few weeks, when he suddenly became very much worse. He suffered frequently from severe chills, amounting to rigors. He lost flesh; was very languid and tired; but managed to struggle on with his work as a shoemaker. He sometimes noticed a weakness of his right eye, which seemed liable to styes.

Dropsy then showed itself in the legs, gradually passing into the body and face. For a week he was scarcely able to breathe, propped up with pillows; but he was able soon after to resume his business.

At this time he noticed toothache and neuralgic pains in the right side of his face, a peculiar numbness of the right cheek at meal times, and stiffness of the upper jaw, as though it did not belong to him. He had also a peculiar pinched-up, contracted feeling of the lower lid, nose, and cheek. There was evidently pressure upon the trunk of the superior maxillary nerve; right.

After the dropsy subsided, both lids remained swollen. He was next seized with a most acute pain on the right side of the apex of the head, specially marked at one spot, where it seemed to perforate the skull; it was accompanied by severe neuralgia of the nose and forehead, which resisted all treatment (ophthalmic nerve). Double vision was then noticed, till the lid of the right eye began to drop and covered the sight (motor oculi).

About six weeks from this time he discovered, on raising the lid, that the right eye was quite blind; and he went to the Bradford Eye Institution. Here it was observed that there was a considerable submaxillary swelling on each side, especially right. No prominence of the eyeball was noticed. Galvanism produced no effect on the ptosis. There was absence of sensation on the forehead, upper part of the head and cheek, on the right side. His head felt very heavy on the pillow, and there were severe pains on both sides of the neck, as well as acute pain in the face and head. The ear was not affected, nor the lower jaw, except mechanically by the submaxillary swellings. He had taste, but he could not enjoy his food.

A fortnight after reaching Bradford, the eye was noticed to protrude. He remained there two months, and when he left he could not close the lids over the globe. He says the tumour was considered to be malignant at the base of the skull, pushing forward the eye.

After a short time he came under the care of one of the surgeons in the Leeds Infirmary. At the commencement of 1885 the eye was continuing to protrude, and was now itself the main seat of the pain. There was a large red mass over the lower lid (conjunctival chemosis), and there was tremendous swelling under each jaw. The surgeons had differences of opinion as to the character of the disease, and no special treatment was adopted for about a month. He was then most energetically treated by mercurial medicines and inunctions; by it his teeth were loosened and his mouth ulcerated: he could take nothing solid for five weeks. He says, whilst he was in the Leeds Hospital he became totally blind, but the sight soon returned to the left eye. The proptosis of the right became more marked, and the eye seemed to lie out upon the cheek. Three months after his admission, removal of the eye was recommended, and would have been done, but that his surgeon fell ill and the patient went home for a change. He says that nothing gave way, but that the swelling gradually subsided *without discharge,* so that when he returned to the hospital they scarcely knew him. He had been living mainly on suet and milk. After re-admission at Leeds, the lids were sewn together with silver wire, in order to protect the cornea, which was becoming cloudy. He admits that at this time there was so much discharge that it required syringing out, and attributed it to inflammation of the eye resulting from the stitches. He seems certain there was no discharge from the eye before. (Had the discharge commenced in the fortnight he was at home, the subsidence of the swelling would be explained by suppuration of part of the neoplastic tissue.) The stitches were removed from the lid at the end of three weeks, and the cornea had improved. He remained an out-patient at Leeds for two months, taking iodide of potassium.

In the middle of 1885 he came under my care in the Infirmary, Bristol. He was very thin and pale. He denied any history of syphilis, and said that mercury always did him harm, but that he felt better when taking iodide of potassium. All his organs were healthy; there was no anasarca. The left eye was quite healthy; the right was prominent, but could be completely covered by the lids, which, however, opened very imperfectly. The elevator was not paralysed. There was a distinct hard non-pulsatile nodule, as large as a nut, along the lower floor of the orbit, which pushed up the eye and was lost in swelling of the conjunctiva, which was very injected and swollen over it. The eye was only capable of the slightest movement in any direction; the cornea showed leucomata, but was not inflamed. There was a slight but definite fulness below the malar bone and zygoma, and above the zygoma in the temple; but the movements of the jaw were not impaired. There was evidently a growth in the spheno-maxillary fissure, causing œdema merely of the temporal and pterygoid regions (for there was no impairment here of nerve or muscular function), surrounding the optic nerve and muscles of the eye, and passing forwards along the anterior extremity of the fissure to the lower wall of the orbit; but the pathology of the growth was uncertain.

I still have the case under observation. The swelling has gradually subsided, but at uncertain times it varies in size. Partial anæsthesia of the implicated nerves still remains, and there is no movement of the eye. For some time there has been no pain. From what I have observed of the growth under anti-syphilitic remedies, I feel sure it is not syphilitic, and the man has never shown any other symptom of this disease. I have never trusted myself to make an incision along the tumour, which is still as large as a nut at the lower wall of the orbit; but I sometimes feel uncertain if it is a hydatid, and I should be glad of an excuse for an exploratory incision. The eye is perfectly blind with atrophy of the disc, seen with difficulty through hazy media.

I may add that on October 21st, 1886, Messrs. Critchett and Juler exhibited a case at the Ophthalmological Society, resembling this one in many respects. I mentioned my case at that meeting, but it has not before been published as I was anxious to keep it longer under observation.

